# Dr. Enoch Vine Stoddard

**Published:** 1908-07

**Authors:** 


Dr. Enoch Vine Stoddard, of Rochester, N. Y., died at his home in that city June 6, 1908, after a prolonged illness, aged sixty-seven years. He was a member of the several local, state and national medical societies; he served in the Civil War as assistant surgeon of the 65th N. Y. volunteer infantry, in which regiment he was also promoted surgeon but was not mustered as such; he was commissioner of health of Rochester for two years and was president of the State Board of Charities for five years; he was professor of materia medica and therapeutics in the University of Buffalo from 1873 to 1890, and thereafter until his death emeritus professor in the same chair; he was a companion of the Military Order of the Loyal Legion of the United States, being a member of the commandery of the state of New York.

